# Evaluation of the Performance of Low-Cement Strain-Hardening Cementitious Composites Containing Desert Sand and Ground Scoria Rocks

**DOI:** 10.3390/ma16175896

**Published:** 2023-08-29

**Authors:** Galal Fares, Mohammad Iqbal Khan

**Affiliations:** Department of Civil Engineering, College of Engineering, King Saud University, P.O. Box 800, Riyadh 11421, Saudi Arabia; galfares@ksu.edu.sa

**Keywords:** desert sands, strain-hardening properties, PVA microfibers, BSE microstructure-based technique of analysis

## Abstract

Fine aggregates are the main ingredients that control the success of the preparation and performance of strain-hardening cementitious composites (SHCCs). Worldwide deserts can be used as eternal sources of fine aggregates for the preparation of SHCCs. Arabian Peninsula desert sand spreads over the largest desert area in the world, covering an area of 2,300,000 km^2^ among the Arabian Gulf countries. White and dune desert sands were procured for use in this study. The morphological structure is important in selecting the appropriate sand for use in the preparation of SHCCs. The utilization of microfibers such as polyvinyl alcohol (PVA) has become common practice for the preparation of SHCCs. The presence of desert sand is proven to enhance the dispersibility of PVA due to its spherical structure, which alleviates the friction among the ingredients forming SHCCs. Two mechanisms are defined under the tensile force at the interface of microfibers and natural sand, namely, a strong frictional force leading to rupture or a weaker force causing pullout. The synergy between fibers and fine aggregate grains depends on their surface characteristics, which can be modified using different types of mineral admixtures. In this research, the alignment of microfibers as an indication of the quality of dispersion could be evaluated using a proposed approach based on an advanced technique of microstructural analysis. PVA dispersion and its relation to strain-hardening properties are visually correlated to the surface interaction of the mineral admixture and dune sand. The microdurability and cost effectiveness of SHCCs could be assessed using the proposed approach, as depicted by the results obtained in this research work.

## 1. Introduction

The applicability of engineered cementitious composites, also known as strain-hardening cementitious composites (SHCCs), in our daily lives has not reached the same frequency of production and practice as in the case of normal concrete (NC) [1]. The main purposes of using SHCCs are to overcome the brittle failure of NC and provide a high level of ductility, which is required in many structural applications. SHCCs exhibit strain-hardening response characteristics under tensile and flexural stress, with multiple microcracks forming in the cementitious matrix [2]. Similar ingredients used in NC are also used in SHCCs, excluding coarse aggregates, to maintain and control the cementitious matrix’s fracture toughness and avoid the fiber’s balling effect. Fly ash plays an important role in defining the fracture toughness of composites. It is usually used in high volumes along with Portland cement to control the progress of the mechanical properties [3,4,5,6]. The mix design and the target mechanical properties necessitate the utilization of silica fume or slag [7,8,9]. Fine quartz aggregates, such as white and dune sands, can be combined with an optimal amount of microfibers. Among the largest deserts on earth, white and dune desert sands spread over some 2,300,000 km^2^ of the area among the Arabian Gulf countries. When handled professionally, they become everlasting sources of fine aggregates for the current and following generations. The use of desert sand as local sand in formulating various high- and ultra-high-performance composites using different fiber types is a relatively new area of study. SHCC preparation using microfibers of polyvinyl alcohol (PVA) has become common practice, with an average content of 2% (volume-based). Other lower contents have also been investigated [10]. PVA is a hydrophilic fiber with a strong hydrogen bond with the hydroxyl groups and presents chemical polarity in the cementitious matrix. The bonds formed between the fibers and the cementitious matrix become stronger than the strength of the fibers, forming a fibro-cementitious composite. A lateral force generated during a direct tensile test accelerates the rupture of bridging fibers while delaying fiber pullout. Variously tailored PVA fibers should be assessed to adopt particular design strategies and behaviors. The mechanism of accomplishment of the strain-hardening response is greatly affected by the interaction along the cementitious matrix/PVA interface [11]. Fine sand plays an important role and significantly affects the interaction mechanism. Fine sand affects not only the fracture toughness of the matrix but also the efficiency of PVA fiber dispersion and consequently the overall quality. The surface structure of sand significantly affects the strain-hardening response of SHCCs under tension [12]. It influences the dispersing power of PVA microfibers and their frictional force concerning the matrix. The type of fiber failure, either rupture or pullout, is also defined by the fiber’s mode of action under tension. The key objective of the current work is to highlight the significance of the sand’s surface structure and its compatibility with supplementary cementing materials in the preparation of cost-effective and microdurable SHCCs. The production of SHCCs demands fly ash (FA) as a hydration regulator, cement, fine aggregate, and microfibers such as PVA microfibers as essential ingredients. Scoria rocks, also known as natural pozzolan (NP), are abundant in the Arabian Peninsula along the Arabian Shield on the Red Sea’s eastern shore. NP extracted from this region has been extensively investigated as a cementitious material [13]. The amorphous nature of glassy materials such as natural pozzolan (NP) from the ground scoria rocks abundantly available in the Arabian Peninsula enhances its latent pozzolanic properties as a hydration regulator to play a role similar to that of FA, but with improved interstitial properties [11,14,15,16]. Optimized physical properties, such as grain size or specific surface properties, lead to the successful use of NP as an alternative cementitious material for cement. When optimally ground, the glassy nature and silicate structure of NP make it an ideal material to minimize the frictional force normally created between PVA and the cementitious matrix due to the hydrophilicity of PVA. The presence of NP also enhances microfiber slippage under tension, which leads to a quicker formation of microcracks with steady propagation under controlled crack opening. The incorporation of NP improves the matrix–fiber interfacial strength, which controls the slip hardening of PVA fibers. Advanced applications of SHCCs are now being developed and extensively used in repairing, retrofitting, and 3D printing materials [17,18]. Higher classes of SHCCs can also be prepared. An ultra-high-strength ECC mix with superior properties was prepared using silica fume and a hybrid combination of polyethylene and steel fibers, with an average crack opening of 72 µm at a strain capacity of 5.2% under tensile strength [19]. The mechanical properties of this advanced type of SHCC were further investigated [20]. In these studies, PVA was excluded from the mix composition. However, in this study, due to the SHCC’s moderate properties, PVA was required to use, investigate, and accordingly estimate the fiber orientation as it affected the mechanical properties. The orientation and counting of the number of PVA fibers per cross-sectional area are beneficial to qualifying the mix composition. Using automatic image processing workflows and simulations using computational fluid dynamics has enabled the derivation of fiber orientation [21]. The use of nanosilica and advanced materials in the production of SHCCs for printing applications with the effects of curing conditions, self-healing, and anti-cracking properties has been reported by many researchers [22,23,24,25,26]. Furthermore, steel corrosion and bonding of steel rebar in the presence of SHCC manufactured using sea sand and seawater are investigated [27,28,29]. The unattainability of these methods makes them difficult to conduct for many researchers. A simple and practical technique is proposed and evaluated in the current work. Accordingly, the novelty and significance of the current work emerge from the easy-to-carry method and high-precision data that can be correlated to the mechanical properties of the SHCC mixes. This approach can be easily used for accurate prediction of the results and the intensive use of SHCC in real-world applications.

## 2. Experimental Investigation

### 2.1. Materials

**Figure 1:**
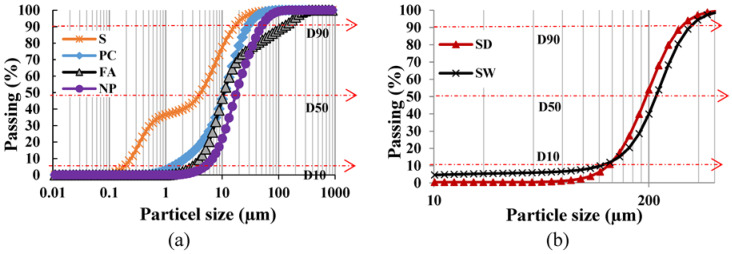
PSD analysis of materials, (**a**) fine powder and (**b**) fine aggregates used in the preparation of SHCC mixes.

**Figure 2:**
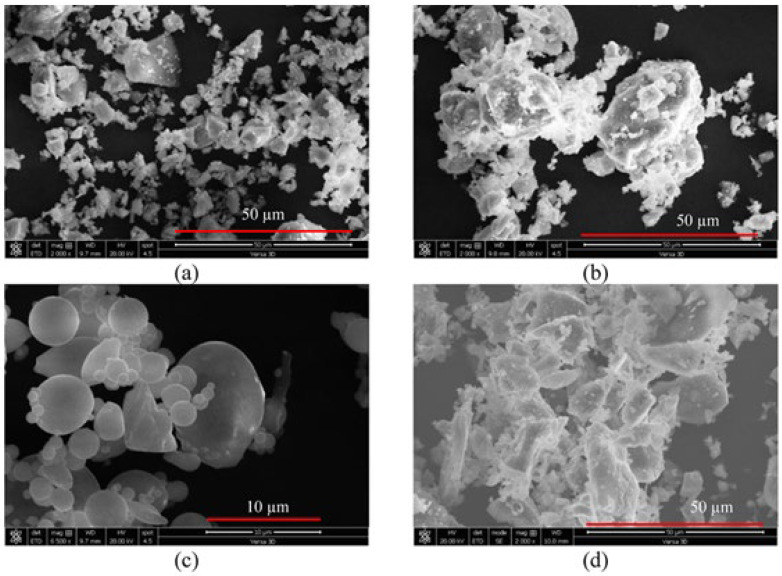
Photomicrograph of fine materials (**a**) S, (**b**) PC, (**c**) FA, and (**d**) NP.

**Figure 3:**
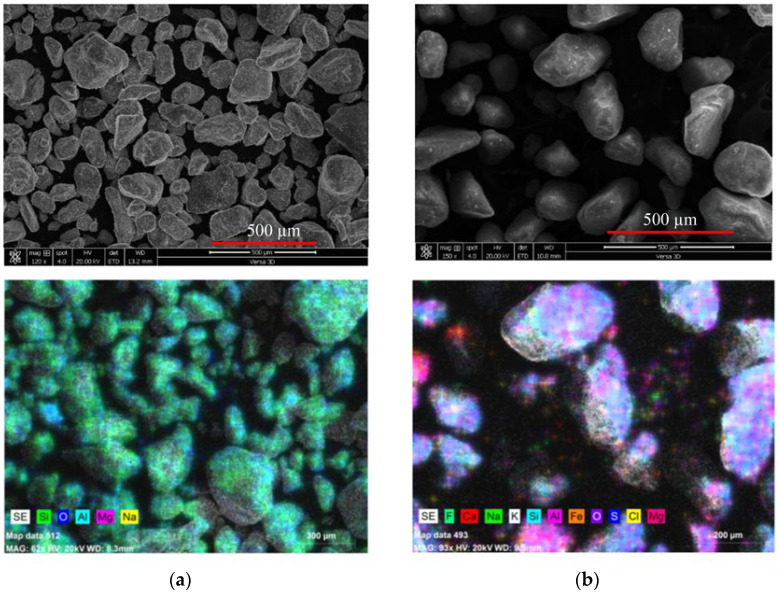
Photomicrograph of natural (**a**) SW and (**b**) SD desert sands.

Different powders forming the main binder for the cementitious matrix of SHCC were used during its preparation, satisfying the requirements of ASTM C150 specifications. There are two approaches to blending Portland cement (PC), either with fly ash (FA, class F), slag (S), or ground scoria rocks as natural pozzolan (NP). The chemical analysis of these powders is given in Table 1. This table reveals an evident similarity in the composition between PC and S. The laser scattering particle size distribution (PSD) analysis of the powder was conducted on a Partica LA 950V2 from Horiba. The median particle size (D50) is one of the main distribution parameters in this test. PSD analysis is shown in Figure 1a. D50 values of 11, 4, 11, and 17 µm were found for PC, S, FA, and NP, whose specific gravities were 3.14, 2.9, 2.3, and 2.8, respectively. Two types of sand were procured east of Riyadh, Saudi Arabia. They are identified as white and red dune sands (SW and SD, respectively) sieved over a 300 µm sieve before use. PSD analysis has shown that the D50 values for WS and DS were 229 and 200 µm, respectively, as demonstrated in Figure 1b. A dual-beam field-emission Versa 3D scanning electron microscope (SEM) was used to study the microstructural analysis of fine materials and sands. SEM analysis of fine materials is shown in Figure 2. According to both Figure 1 and Figure 2 where S has the finer particles and NP has the coarsest particles, the PSD analysis and the SEM analysis are in good agreement. The X-ray energy dispersive spectroscopy analyzer attached to SEM (SEM-EDS) was used to reveal the surface composition of each type of sand (SW and SD), as shown in Figure 3a,b, respectively. From this analysis, it is concluded that both sands have a spherical structure and different surface chemistry. The spherical structure differs from the angular one in improving the rheological properties of SHCC mixtures. EDS analysis illustrates the main surface elemental structure difference between SW and SD. The surfaces of SW grains are saturated with a silicate network. In contrast, those of SD grains are a mix of calcosilicate networks, indicating the presence of various elemental compositions, namely, impurities. This difference is expected to affect the matrix and its relationship with PVA.

**Figure 4:**
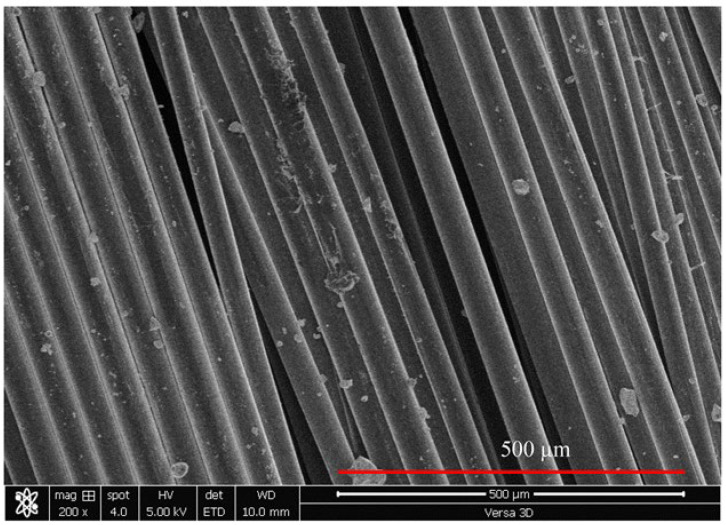
Photomicrograph of PVA fibers.

This study used a polycarboxylic ether-based (PE) superplasticizer as the main superplasticizer. PE has a water content of 64% and a solid content of 36% with a density of 1.1 g/cm^3^. PVA represented microfibers whose length and median diameter were 12 and 40 µm, respectively. Accordingly, PVA had an aspect ratio of 266. The microstructural analysis of PVA microfibers using SEM is shown in Figure 4.

### 2.2. SHCC Mix Proportions

The optimized SHCC mix had an optimized PVA content of 2.1% (volume-based), with the mix composition detailed in Table 2. The optimized PE dosage range of 2 to 2.3 kg/m^3^ was applied in the investigated mixes where the elevated dosage was used with a high replacement level.

### 2.3. Mixing Procedures and Testing

**Figure 5:**
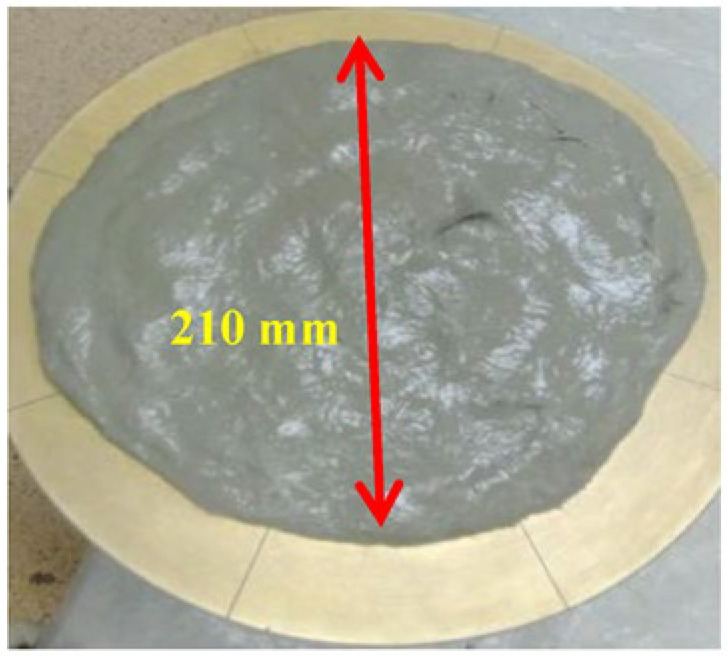
Flow table value of SHCC mixtures.

**Figure 6:**
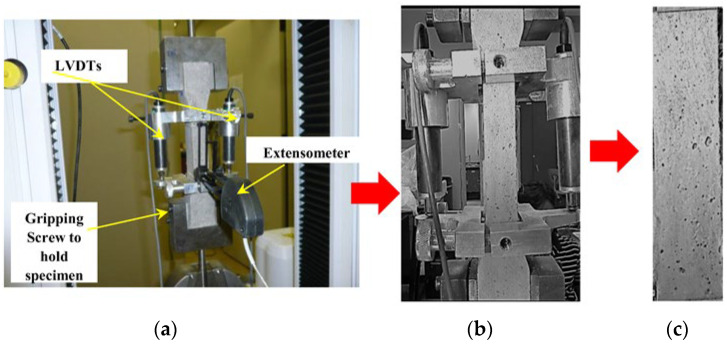
Direct tension testing. (**a**) Testing setup, (**b**) crack propagation face, and (**c**) isolated face for analysis.

A Hobart mixer was used in the preparation of SHCC mixes. The mix started with homogenizing the optimized dry materials in the mixer bowl at low speed, as it has two speeds, for a total of 2 min. A homogenized mix of water and optimized PE was then stirred in an external container and poured over the dry materials in the bowl under low-speed mixing until achieving the target workability. The PVA microfibers were added at high speed until satisfying apparent efficient fiber dispersion was achieved in about 2–3 min. The average spread-flow value of the optimized mixtures has a flow value higher than 200 mm using the flow table test as an indicator for workability, as shown in Figure 5. For tensile properties, dumbbell-shaped molds with a dimension of 40 mm × 40 mm × 240 mm were used for the uniaxial monotonic tensile loading test, as shown in Figure 6. After demolding samples, they were wrapped in plastic sheets to isolate them from the surrounding environment until the testing ages of 7 and 28 days. Uniaxial tensile strength along with ductility was evaluated under a quasi-static strain rate of 3.1 × 10^−5^ s^−1^ using the Instron machine model 5500 connected with a built-in digital data recorder for the applied tensile load and resultant displacement.

### 2.4. Fiber Orientation Technique Using BSE Compositional Imaging

**Figure 7:**
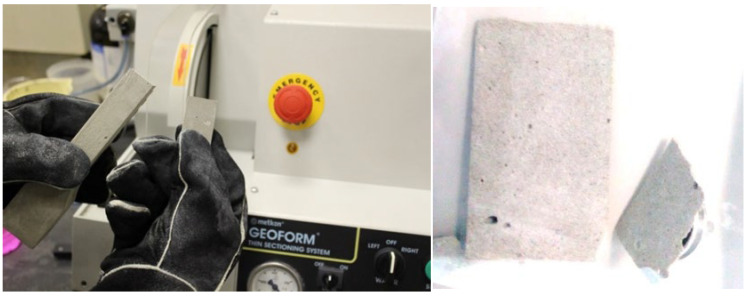
Slicing machine and the obtained micro-sized slice for SEM-EDS analysis.

**Figure 8:**
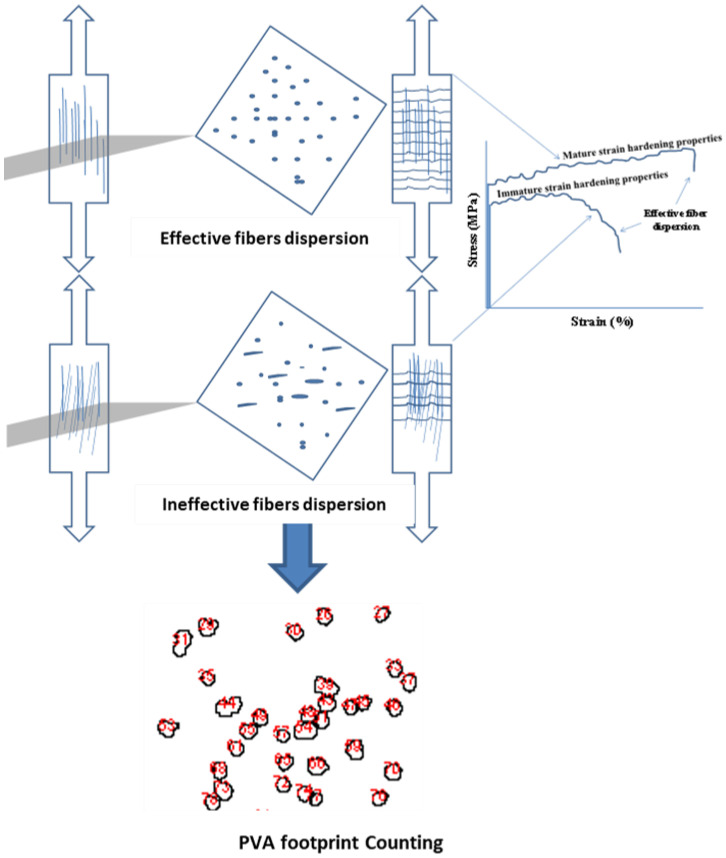
Schematic representation of the current approach and its efficiency in interpreting the strain-hardening properties.

The main concept of this technique relies on the compositional difference among elements in the SHCC sample as revealed by high-resolution images of these elements using a backscattered electron (BSE) detector available in the scanning electron microscope. To improve imaging quality, the middle part of the dumbbell-shaped samples should be cut into cross-sections using a diamond blade cutter in the grinding and cutting machine Geoform, as depicted in Figure 7. The axially distributed PVA microfibers, mainly composed of carbon, will appear as scattered points, while the inclined ones will appear as lines, as demonstrated in Figure 8. In SEM-EDX, the element can be optionally identified by any defined color. The nature of PVA/matrix bonding can also be simply identified through the same technique. Using any of the available image analysis techniques helps identify the number of fibers over a 16 mm^2^ cross-sectional area.

## 3. Results and Discussion

### 3.1. Tensile Stress–Strain Properties of SD with FA and GGBFS

**Figure 9:**
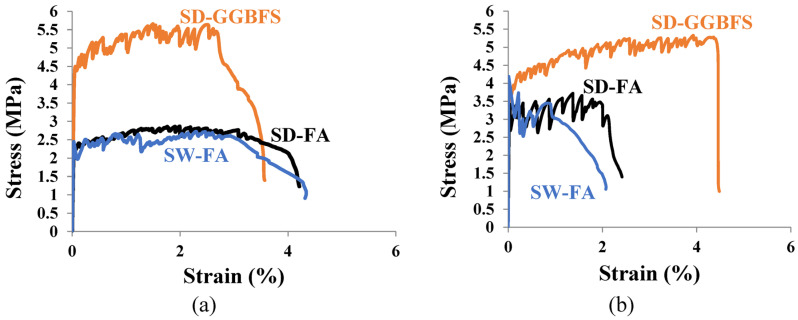
Stress–strain curves of SHCC mixtures at (**a**) 7 days and (**b**) 28 days.

**Figure 10:**
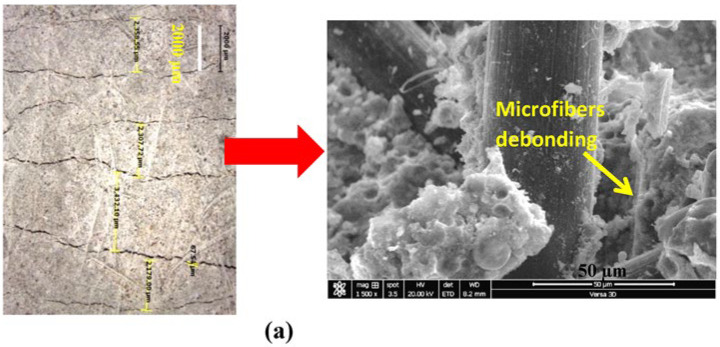

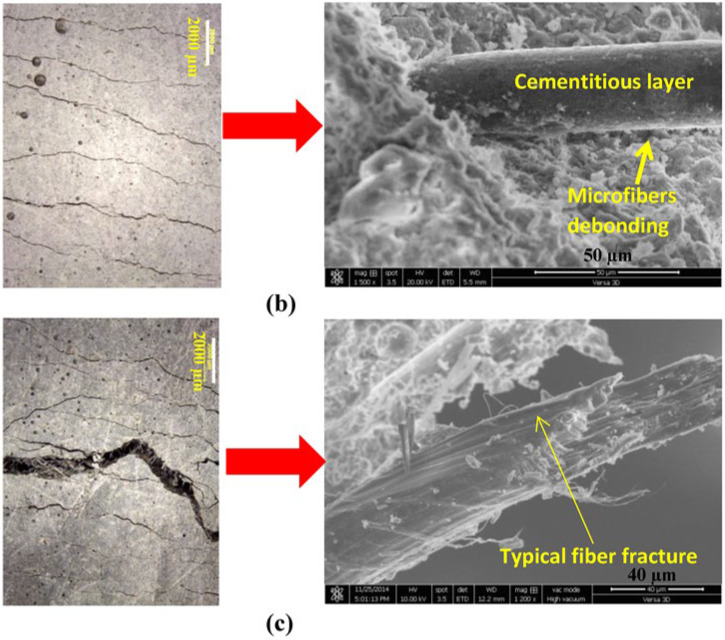
Effect of mix composition on cracking pattern and fiber pullout mechanism in (**a**) SD-FA, (**b**) SD-GGBFS, and (**c**) SW-FA mixtures.

The effects of the composition of the cementitious matrix and aggregate type over different cutting ages of 7 and 28 days on the ductility of SHCC mixtures (S1–S3) are demonstrated in Figure 9a,b, respectively. The lab study shows that both sands within the same mix design provide different results at 28 days. The mixture with DS provides improved properties attributed to the surface composition of its grains, as shown in Figure 3a. It is also clear from Table 1 that there is a similarity in the chemistry of both PC and GGBFS. As a result, and to improve the properties of the SHCC mixture with DS even further, FA is completely replaced by GGBFS in the new mix design. The results at seven days have shown that both SHCC mixtures with SW and DS have given similar results at seven days with FA with low tensile strength and improved ductility, namely a strain capacity beyond 3%, as presented in Figure 9a. This was attributed to the slow pozzolanic activity of FA, which could not impose the main difference between sands. However, the SHCC mixture with DS and GGBFS has doubled the tensile strength due to the early hydraulic properties of GGBFS but with a slightly low strain capacity, as clearly shown in Figure 9. At a curing age of 28 days, the pozzolanic properties of FA were initiated along with cement hydration products. The ultimate tensile strength went beyond 3 MPa but with reduced ductility, as shown in Figure 9b. This was attributed to the fiber/matrix interface properties, which are investigated using SEM-EDX analysis. Due to the hydrophilic properties of PVA fibers, a strong bond between the fibers and matrix is expected, which can be improved by modifying the mix design and components of the cementitious matrix. The SHCC mixture with DS has improved tensile properties with multiple cracks and limited strain-hardening properties, namely a strain capacity of about 2%, which is nearly negligible in the SHCC mixture with SW. It is worth remembering that the chemical similarity between PC and GGBFS is matched with the surface composition of DS, namely a Ca-rich surface identified as a calcosilicate network, as inferred from Table 1 and Figure 3b. However, Figure 9b shows that the tensile strength did not change while the strain capacity improved beyond 4% with strain-hardening properties and a crack opening of less than 60 m. A microstructural analysis would help interpret these results. The microstructural analysis of the samples near microcracks reveals the fiber/matrix interaction mechanism, as shown in Figure 10. A fiber pullout is observed in the mixtures S1 and S3, where SD sand and FA and GGBFS were used, as shown in Figure 10a,b. A notable fiber rupture is observed in mixture S2, as shown in Figure 10c. Therefore, SD sand is considered the optimum sand for use in the preparation of SHCC.

At 28 days, the average crack spacing was approximately 2.6 mm while the average crack opening was approximately 70 µm. The cracking information of the mixtures S1 to S3 is reported in Table 3. The cracking patterns indicate that SHCC (S1 to S3) mixtures have reached a steady state of constant crack opening, which is prevailing in an obvious manner. At 28 days, the fracture toughness seems to have increased due to the progress in hydration, which represented another challenge to resolve within this study to improve the strain-hardening properties to their maximum. It is therefore concluded that SD sand is considered the optimum sand for efficient use in the preparation of SHCC. GGBFS is compatible with SD and much more effective than FA in SHCC. However, GGBFS is not always available in many countries and it is not cost-effective. Alternative local materials, such as ground scoria rocks as natural pozzolan, are now strongly recommended to partially replace FA. This approach should reduce the overall cost of SHCC preparation when successful.

### 3.2. Tensile Properties of SD-FA-S-Based System

**Figure 11:**
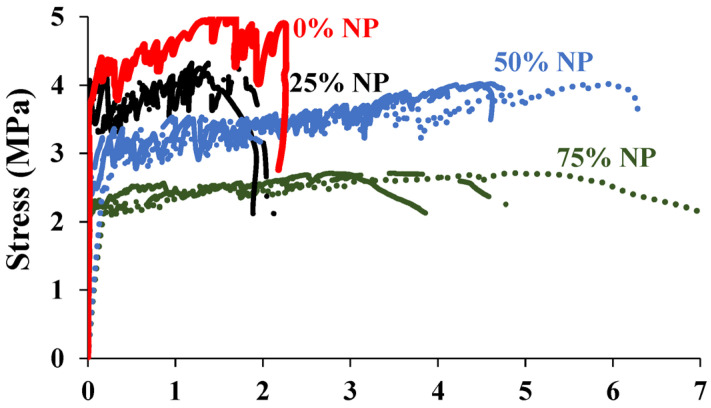
Tensile properties of mixtures with 0% NP at 7 and 28 days.

**Figure 12:**
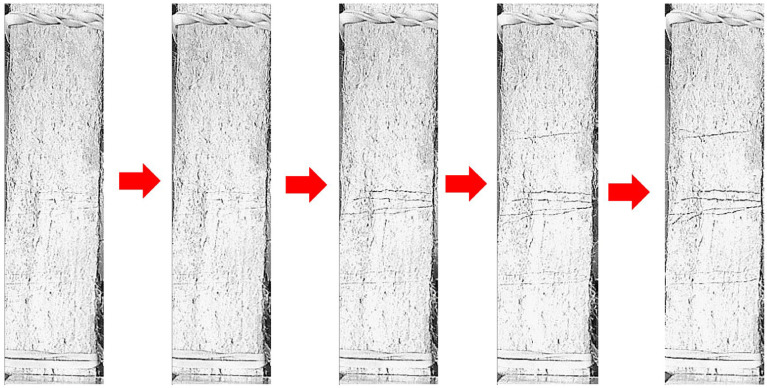
Progressive crack propagation of the control mixture until complete failure (0% NP).

**Figure 13:**
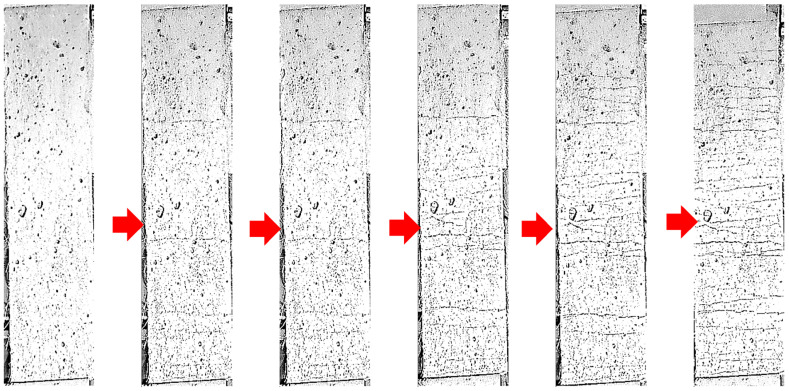
Crack propagation of the mixture containing NP until complete failure (50% NP).

This paper is the first to introduce ground scoria rocks into SHCC within the field of SHCC preparation. The results of the SHCC samples (S4–S7) with NP replacement levels of 0, 25, 50, and 75% NP are shown in Figure 11. The strain capacities of the samples with 50 and 75% NP show more than 6% improvements compared to the samples with 0 and 25% NP, FA, and GGBFS. Further, cracking measurements were conducted for each sample and the average results are shown in Table 4. The crack spacing and crack opening have significantly reached the minimum values in the mixtures with 50 and 75% NP, compared to the samples with 0 and 25% NP. As well, the number of cracks increased in the samples with 50 and 75% NP, as presented in Table 4. The replacement level of 50% NP provided the optimum strain hardening and tensile strength response. The monitored progressive crack propagation until failure in the control mixture with 0% NP compared to the optimum sample with 50% NP is shown in Figure 12 and Figure 13, respectively. It was evident that the number of cracks had reached its maximum count of more than 50 in the mixture with 50% NP.

### 3.3. Optical Microscopic and SEM Analyses

**Figure 14:**
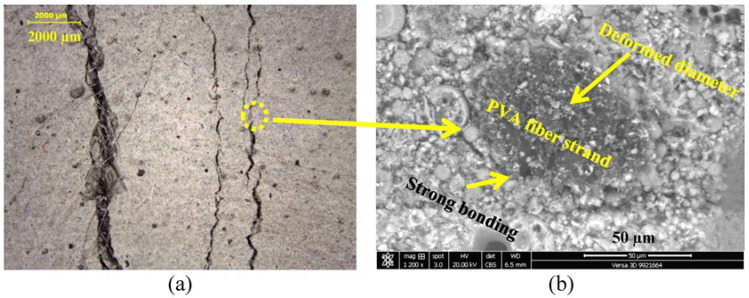
Cracking patterns along with microstructural analysis at fiber/matrix interfacial transition zone (FM-ITZ) of SHCC mixture with SW and FA as a pozzolanic material using (**a**) optical microscope and (**b**) BSE analyses.

**Figure 15:**
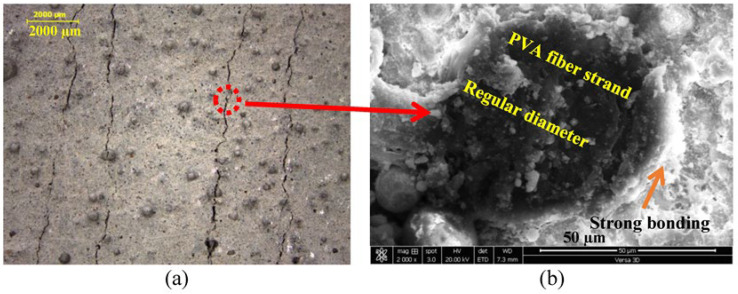
Cracking patterns along with microstructural analysis at fiber/matrix interfacial transition zone (FM-ITZ) of SHCC mixture with DS and FA as a pozzolanic material using (**a**) optical microscope and (**b**) BSE analyses.

**Figure 16:**
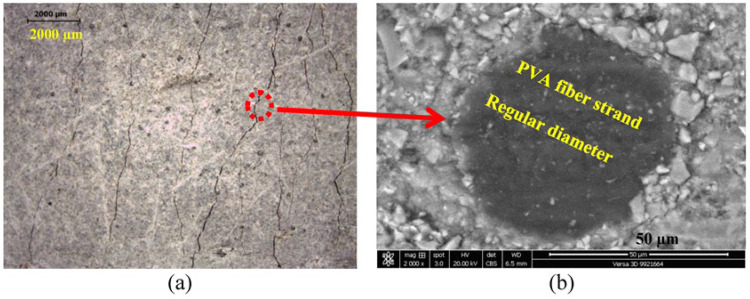
Cracking patterns along with microstructural analysis at fiber/matrix interfacial transition zone (FM-ITZ) of SHCC mixture with DS and S as a cementitious material using (**a**) optical microscope and (**b**) BSE analyses.

In this part of the SEM-EDX analysis, as described earlier, thin sections were taken from tested samples (S1–S3) after optical microscopic investigation at 28 days. The results of this analysis are shown in Figure 14, Figure 15 and Figure 16. The optical microscopic analysis has revealed that the SHCC mixture S2 with SW and FA has a low cracking pattern, as depicted in Figure 14a. The mixture shows a large crack width, indicating the reduced efficiency of PVA fiber due to low dispersion and high bonding with fibers, which led to fiber rupture, as shown in Figure 10c. On the other hand, the SHCC mixture with SD and FA has the characteristic strain-hardening properties of multiple cracking and a higher crack width than 60 µm, as demonstrated in Figure 15a. Similarly, the SHCC mixture with SD and GGBFS has the characteristic strain-hardening properties of multiple cracking and a fine crack width of less than 60 µm, as shown in Figure 16a. The cracking system can also be taken as a reflection of the quality of PVA fiber dispersion with improved properties at the interfacial transition zone (FM-ITZ) at the fiber/matrix region, as shown in Figure 14b, Figure 15b, and Figure 16b. An SEM microstructural analysis has enabled the characterization of FM-ITZ in mixtures with SW and SD with either FA or GGBFS. At FM-ITZ, the nature of the bonding between fiber and matrix can be qualified. In the SHCC mixture with SW and FA, PVA fiber has a deformed perimeter; it has lost its sphericity, namely its diameter, as an indication of the strong bonding with the matrix that leads to an elevated frictional force, as confirmed in Figure 10b. In the mixture with SD and FA, the PVA fiber has lower deformation in its diameter as an indication of lower bonding with the matrix compared to the mix with SW and FA, as shown in Figure 10c and Figure 14b. However, Figure 15b shows the existence of strong bonding with the PVA fiber due to its hydrophilicity. Still, it seems to have a lower frictional force than the case with SW and FA due to the nature of the hydration products at FM-ITZ between SD and FA, as clearly shown in Figure 10a and Figure 15b. In the mixture with SD and GGBFS, the PVA fiber has a very low deformation in its diameter compared to other mixtures of SW and SD with FA, as shown in Figure 10b and Figure 16b. The hydration products at FM-ITZ seem to be completely different from those found in the samples of SW and DS in the presence of FA. Therefore, the hydrophilicity of the PVA fiber has been modified due to the chemical nature of the SHCC ingredients. The bonding with the matrix is expected to be adjusted while the friction force is reduced, along with significantly improved PVA fiber dispersion. These claims can be concluded from the results shown in Figure 9, Figure 14, Figure 15 and Figure 16. Controlling the crack width and spacing is one of the tools to improve the microdurability of SHCC mixtures. The use of a self-healing agent is recommended for extending the microdurability of SHCC mixtures.

### 3.4. BSE Compositional Imaging

**Figure 17:**
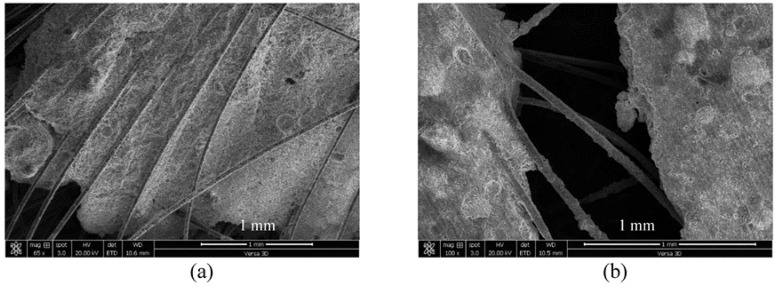
SEM photomicrograph of (**a**) well-oriented fibers and (**b**) randomly distributed.

**Figure 18:**
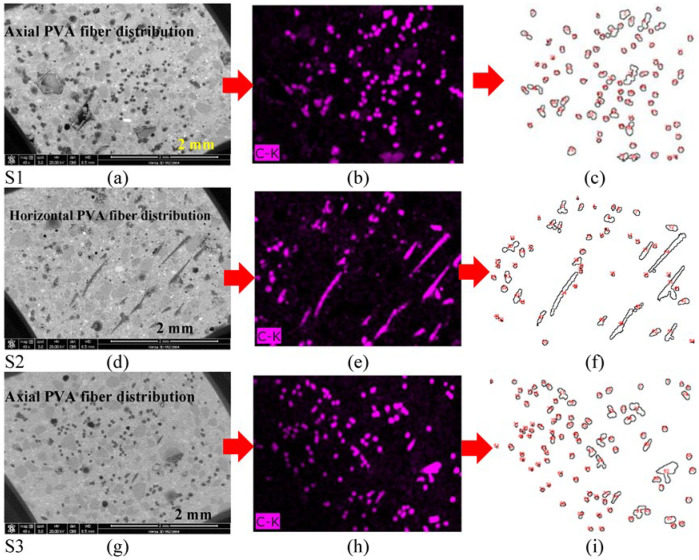
Photomicrographs of (**a**,**d**,**g**) backscattered electron detector analysis using (**b**,**e**,**h**) SEM-EDX elemental mapping and (**c**,**f**,**i**) counting of fibers footprint using image analysis for the samples (S1–S3), respectively.

This part of the analysis uses BSE compositional imaging to determine the fiber orientation in SHCC samples. The same samples used in evaluating FM-ITZ using the samples’ thin sections were also investigated using the available BSE technique, as described in Figure 7 and Figure 8. Figure 17 illustrates the typical PVA microfiber distribution, highlighting how the pattern of the fiber footprint depends on whether the microfibers are well-oriented or randomly distributed. The results of these analyses for the samples (S1–S3) are shown in Figure 18. The footprint of PVA fibers is the highest in S3followed by S1, while it is the lowest in S2 where it contains WS and FA. In addition, the orientation of the fibers is side-aligned, as opposed to S1 and S3 where the fibers are vertically orientated on the cross-sectional area, as illustrated in the figure. These results confirm the data obtained in Figure 10, Figure 14, Figure 15 and Figure 16. These data are in agreement with the study that investigated the effect of the flow direction on the fiber orientation [30,31]. They concluded that the flow-induced casting leads to a substantial improvement in the alignment of fibers in the casting direction, significantly improving the mechanical properties.

### 3.5. PVA Footprint Counting Theoretical Approach

**Figure 19:**
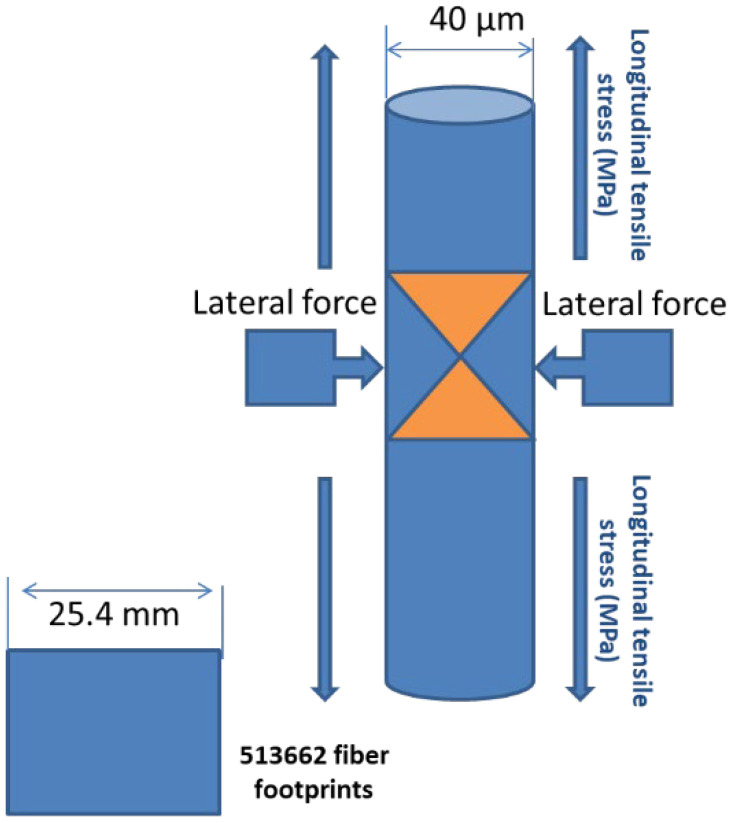
Total theoretical number of PVA footprint to occupy the whole cross-sectional area of the sample.

The average counts of microfibers per area of 16 mm^2^ (cross-sectional) were 92, 54, and 106 for the S1, S2, and S3 samples. The samples in the dumbbell shape have a cross-sectional area of approximately 645 mm^2^. Accordingly, as per the given fiber dimension and the sample cross-sectional area, the total theoretical number of PVA footprints to occupy the whole cross-sectional area of 645 mm^2^ is estimated to reach about 513,662, as demonstrated in Figure 19. Based on these data, the percentage ratio of the actual count to the theoretical count of samples S1, S2, and S3 is given in Table 5.

## 4. Conclusions

The successful production of SHCC with the desired properties can be obtained by optimizing its mix design and constituent ingredients. Fine sand is the main ingredient in retaining the strain-hardening properties of SHCC mixtures. When matched with the cementitious matrix’s chemical composition, the sand’s surface elemental composition becomes a controlling parameter that defines the strain-hardening properties. The type of supplementary cementitious materials is another key player that integrates with both the cementitious matrix and sand to define the nature of the hydration products at FM-ITZ. The quality of FM-ITZ defines and participates in the quality of PVA fiber dispersion, its bonding, and its frictional forces with the matrix. The compatibility of the chemical composition of the main constituents of the SHCC mixture helps to obtain a mixture with strain-hardening properties and a lower crack width. The spacing between cracks and their widths are controlled by using ground scoria rocks as one of the tools to improve the micro-durability of SHCC mixtures. The use of BSE is an efficient tool that enables the evaluation of the quality of dispersion of PVA microfibers.

## Figures and Tables

**Table 1:** Chemical composition of fine materials and their physical properties.

	PC	S	FA	NP
SiO_2_	20.2	34.53	50	43.31
Al_2_O_3_	5.49	14.23	28	15.41
Fe_2_O_3_	4.12	0.66	10.4	12.48
CaO	65.43	41.38	4.4	9.26
MgO	0.71	5.59	1.3	10.10
Na_2_Oeq	0.26	0.21	1.5	3.44
SO_3_	2.41	0.6	0.35	0.06
Loss on ignition (%)	1.38	2.45	4	0.95
Median Grain Size (µm)	11	4	11	17
Specific gravity	3.14	2.9	2.3	2.8
Fineness (m^2^/kg)	373	400	450	280

**Table 2:** The main mix design used in this study (kg/m^3^).

Mix ID	PC	GGBFS	FA	NP	SD	SW	Water	PVA
S1	475	0	582	0	486	0	370	27
S2	475	0	582	0	0	486	370	27
S3	280	840	0	0	520	0	391	28
S4	555	0	666	0	466	0	315	26
S5	416	0	666	139	466	0	315	26
S6	257	0	666	257	466	0	315	26
S7	128	0	666	386	466	0	315	26

**Table 3:** Cracking information after testing for S1–S3 at 28 days.

	Crack Opening	Crack Spacing
Range of measured values	70–200	2.1–3.4
Average value	70	2.57

**Table 4:** Cracking system measurements.

	0–25% NP		50–75% NP	
	Crack Opening (µm)	Crack Spacing (mm)	Crack Opening (µm)	Crack Spacing (mm)
Range of measured values	40–80	0.55–3.73	<50	0.75–1.8
Average value	50	2.08	<50	1.3
Number of cracks	<50		>50	

**Table 5:** PVA footprint counting results.

		S1	S2	S3
Count	Area of 16 mm^2^	92	54	106
	Area of 645 mm^2^	3708	2177	4273
	Effective ratio (%)	0.72	0.42	0.83

## Data Availability

Data are contained within the article.
